# Chi-Squared Detection of Physical Accident Signatures in Nuclear Power Plant Data, with Synthetic Adversarial Stress Test

**DOI:** 10.3390/s26216812

**Published:** 2026-10-28

**Authors:** Mordecai Opoku Ohemeng, Frederick T. Sheldon

**Affiliations:** Department of Computer Science, University of Idaho, Moscow, ID 83844, USA

**Keywords:** nuclear power plant accident diagnosis, anomaly detection, chi-squared residual, Isolation Forest, NPPAD benchmark, cyber-informed engineering, synthetic false-data injection, adversarial stress test

## Abstract

Nuclear-Renewable Hybrid Energy Systems (N-RHES) integrate small modular reactors with intermittent renewable generation to deliver flexible, zero-carbon grid balancing. However, their interconnected cyber-physical architecture remains susceptible to adversarial manipulation of digital instrumentation and control pathways. Advanced consequence-driven methodologies, such as Consequence-driven Cyber-Informed Engineering (CCE), prioritize engineering out high-consequence cyber risks rather than relying solely on post hoc detection. This paper presents a benchmark of lightweight detectors motivated by the CCE analytical workflow, instantiated as an anomaly detection pipeline, evaluated on the Nuclear Power Plant Accident Data (NPPAD) dataset. We compare a classical chi-squared (Mahalanobis distance) detector against an Isolation Forest detector, alongside per-feature z-score and PCA reconstruction baselines. All detectors are evaluated at a matched 1% target false alarm rate (FAR) on a random split of the 302 normal samples, yielding an achieved FAR of 0.98%. Chi-squared achieves an AUC of 0.9989 (95% CI [0.9975, 0.9997]) and a 96.61% sample-level detection rate, significantly outperforming Isolation Forest (AUC 0.9762, 81.79% detection rate; DeLong p<0.001). We do not claim to exceed published deep learning baselines, because their evaluation protocols differ substantially. Per-accident analysis shows all 17 accident types are detected at the scenario level, 15 of 17 exceed 95% sample-level detection, and 14 of 17 achieve 100% sample-level detection, with load rejection (70.6%) and rod insertion (92.5%) as the exceptions. To bridge the gap between physical accident detection and the adversarial threat model, we introduce a synthetic false-data-injection stress test. Under bounded sensor drift at the stealthy attack band (0.5σ), chi-squared detection remains at 1.5% ± 0.7%, indistinguishable from its 0.98% false alarm rate. This demonstrates that the 96.61% physical-accident detection rate does not transfer to adversarial manipulation, and that the detector ranking changes under adversarial stress. We interpret this as a dataset-specific finding, NPPAD presents large, physically distinct anomaly signatures well-suited to distance-based detection. This work demonstrates methodological transferability and explicitly characterizes the fidelity gap between commodity-platform analysis and hardware-in-the-loop validation.

## 1. Introduction

Critical infrastructure increasingly depends on cyber-physical integration to optimize operational efficiency and grid reliability. Among emerging architectures, Nuclear-Renewable Hybrid Energy Systems (N-RHES) represent a paradigmatic configuration in which thermal energy from advanced nuclear plants is dynamically shared with electrical grids, hydrogen production facilities, and renewable smoothing applications [1]. While these configurations maximize asset utilization and support decarbonization objectives, they simultaneously expand the attack surface available to persistent adversaries.

The security of cyber-physical systems (CPS) more broadly has become a central research priority. Recent work has demonstrated that the deep integration of computational and physical processes introduces vulnerabilities to intelligent, pattern-aware attacks that can destabilize operations [2,3].Recent surveys of CPS security emphasize that modeling attacker capabilities and designing resilient architectures are equally critical to detection-based defenses [4,5]. False-data injection (FDI), denial-of-service, and stealthy sensor drift are recognized as prominent threats to CPS integrity, motivating both detection and resilient control architectures [6,7].

The response to this expanded threat landscape is not by pursuing complex detection algorithms, but by developing structured methodologies that embed security into system design and operational planning. At Idaho National Laboratory (INL), Consequence-driven Cyber-Informed Engineering (CCE) has emerged as a foundational framework for identifying and mitigating high-consequence cyber risks in critical infrastructure [8]. The CCE methodology proceeds through four phases: (1) identifying High-Consequence Events (HCEs) that would produce unacceptable physical, economic, or safety outcomes; (2) analyzing the systems and functions whose compromise could enable those events; (3) adopting an attacker’s perspective to trace pathways to the HCE; and (4) deriving engineered mitigations that eliminate or substantially reduce the risk rather than merely detecting its exploitation [8].

Complementing CCE, Security-Inclusive Model-Based Systems Engineering (MBSE) in projects such as the MARVEL microreactor, integrating STPA (System-Theoretic Process Analysis) to identify unsafe control actions during the design phase rather than retrofitting security after deployment [9]. The AGN-201 digital twin demonstrates a complementary approach: streaming real-time operational data, control rod positions, reactor power, and temperature, through LabView into the DeepLynx data warehouse, where Isolation Forest (IFML) anomaly detection identifies deviations such as unauthorized material insertions or unexpected reactivity shifts [10]. IFML’s selection is instructive: it is a lightweight, interpretable, and implementation-friendly algorithm well-suited to resource-constrained environments, in contrast to deep learning architectures whose performance on real nuclear data remains moderate [11].

### 1.1. Detection Methods for Nuclear Anomaly Identification

Anomaly detection in nuclear power plants has been approached through several methodological families, each with distinct trade-offs. The most straightforward approach is fixed-threshold monitoring, where predefined upper and lower limits are established for critical sensors. This approach avoids false alarms but triggers only after a fault has developed to a certain extent, resulting in limited real-time warning capability [12]. More sophisticated statistical methods include Principal Component Analysis (PCA), which has been applied to fault detection in generated data with good detectability and diagnosability [13], and Kernel PCA (KPCA), which achieved 96.38% fault detection accuracy in the chemical volume control system of a nuclear plant [14].

Machine learning approaches have gained prominence more recently. Autoencoder-based methods, including convolutional autoencoders with spatio-temporal attention, have demonstrated higher anomaly detection accuracy on Fuqing PWR simulator data [12]. LSTM and recurrent architectures have been applied to sensor reconstruction and anomaly detection, with hybrid LSTM-CAE models outperforming single-model approaches [15]. Isolation Forest has been applied to marine nuclear power turbine units with accuracy exceeding 94% [16], and combinations of sparse autoencoders with Isolation Forest have shown high accuracy on transient operating conditions [17]. Recent reviews of deep learning for nuclear health monitoring document 99 studies, reflecting the field’s rapid growth [18,19]. A recent systematic review of AI-based cybersecurity in nuclear power plants confirms that while AI is essential for real-time threat detection, the scarcity of labeled attack data and the need for interpretable models remain central open challenges [20].

Beyond nuclear-specific methods, neural-network-based estimation and control frameworks have been proposed for CPS more generally. The work in [3] develops an AI-driven estimator that treats adversarial signatures as latent control signals using Lie-algebraic generators, integrated with a Control Barrier Function safety filter to achieve Uniform Ultimate Boundedness under high-potency adversarial pressure. A complementary line of work addresses computational weaponization, resource-exhaustion attacks that degrade control performance under self-triggered switched systems, through an ISS-based framework [21]. These CPS-general methods motivate our interest in lightweight, interpretable detectors that do not require deep architectures to achieve reliable performance in safety-critical settings [22,23].

Despite this proliferation of methods, several challenges persist. Machine learning models often exhibit sensitivity to noise, limited generalization across operational scenarios, and overconfidence in predictions under uncertainty. Deep learning methods require substantial training data and computational resources, which is problematic in nuclear settings where accident data is scarce and labeled fault samples are rare [24], dimensionality further complicates distance-based methods as feature counts grow. These limitations motivate interest in lightweight, interpretable detectors that can operate effectively with limited training data.

### 1.2. Why Chi-Squared Detection for This Work

The chi-squared (Mahalanobis distance) detector is a classical statistical method. It measures how many standard deviations a sample lies from the normal operating envelope, accounting for correlations between features. It is well-suited to the NPPAD detection task for four reasons.

First, the physical structure of nuclear accident signatures are characterized by large, correlated deviations in multiple operational parameters [25]. A loss-of-coolant accident, for example, produces simultaneous changes in pressure, temperature, and flow that are jointly anomalous even if no single parameter is extreme. The Mahalanobis distance captures this joint deviation naturally through its covariance weighting, whereas univariate methods and tree-based methods that partition features independently do not exploit this structure.

Second, limited training data. The NPPAD normal condition contains only 302 samples. Deep learning methods and complex ensembles typically require far more data to train effectively. Chi-squared requires only the estimation of a mean vector and covariance matrix, which is feasible with 302 samples when regularization is applied. The Ledoit-Wolf shrinkage estimator [26] provides a well-conditioned covariance estimate even when the number of features (96) approaches the number of samples (302). This regularization is standard practice in industrial monitoring when calibration data is limited and sensor correlations are strong [27].

Third, interpretability and regulatory acceptance. In nuclear safety applications, detection methods must be explainable to operators and regulators. The Mahalanobis distance has a clear statistical interpretation as a measure of deviation from the normal distribution, and its threshold has a direct probabilistic meaning (the false alarm rate is the probability of exceeding the threshold under the normal distribution). This interpretability aligns with the nuclear industry’s emphasis on explainable AI and safety-case documentation [28,29].

Fourth, the CCE methodology prioritizes engineering solutions over detection, and the detection component should be as simple and reliable as possible so that engineering resources can be directed toward the protections that cannot be engineered out. Chi-squared’s closed-form nature, deterministic behavior (given fixed training data), and low computational cost make it a natural fit for this role.

### 1.3. Related Works

The Nuclear Power Plant Accident Data (NPPAD) dataset was generated using PCTRAN, a widely used simulator whose predictions have been validated against the Fukushima Daiichi accident progression [30]. The dataset covers 18 operating conditions (1 normal + 17 accident scenarios) with 96 operational parameters sampled at 10 s intervals.

The original publication [31] focused on dataset construction and technical validation rather than detection methods. The authors explicitly created the dataset to fill a gap: no open NPPAD accident dataset existed for benchmarking AI algorithms, and most prior studies used private simulators. The dataset was designed to enable reproducible comparison of anomaly detection and accident diagnosis algorithms.

Subsequent peer-reviewed work has applied deep learning to NPPAD with reported quantitative results. Aseeri [32] evaluates out-of-distribution (OOD) detection on NPPAD using high-openness splits that withhold entire accident families as unknowns. The SCOPE framework achieves run-level AUROC of 0.884±0.051, outperforming standard OOD baselines including Mahalanobis distance (0.820±0.064), ODIN (0.819±0.080), and reconstruction autoencoders (0.803±0.004) [32]. This result is directly relevant to our work: it establishes a peer-reviewed reference point for Mahalanobis-based detection on NPPAD, against which our chi-squared detector can be positioned.

Liu et al. [33] address class-incremental fault diagnosis on NPPAD using a cascaded spatio-temporal network combining LSTM and Transformer components with time-frequency fusion. Their method achieves an average accuracy of 91.36% across five incremental learning stages, representing an improvement of approximately 20.7% over baseline models [33]. Ateya and Salman [34] propose a Physics-Guided Spatio-Temporal Mamba Network with dynamic halting, achieving 99.88% final classification accuracy with 97.1% accuracy within 100 s after fault initiation on a PCTRAN-generated CPR1000 benchmark [34].

A parallel line of work addresses adversarial manipulation of CPS signals directly. The work in [6] propose a physics-constrained optimization framework for detecting stealthy drift perturbations, the same attack class evaluated in our synthetic FDI stress test. Their approach leverages physical constraints to identify drift signatures that evade conventional residual-based detectors. Separately, [7] propose a blockchain-augmented CPS framework to mitigate FDI attacks and improve system resiliency, demonstrating that architectural defenses can complement detection-based methods. These works inform our CCE framing: detection alone is insufficient, and engineered protections (including architectural and cryptographic mechanisms) must be prioritized for high-consequence threats.

These prior studies focus on deep learning architectures and report high classification accuracy on NPPAD. Our work differs in four respects. First, we evaluate lightweight, closed-form detectors rather than deep learning models, motivated by the resource constraints and interpretability requirements of operational deployment. Second, we compare chi-squared detection directly against Isolation Forest configured to match INL’s public AGN-201 deployment [10], providing a controlled comparison between two lightweight methods. Third, we frame the analysis within the CCE methodology, connecting detection performance to the engineering protection principle that CCE prioritizes. Fourth, we connect the physical-accident benchmark to the adversarial threat model through a synthetic FDI stress test, quantifying the gap between accident detection and stealthy drift detection that CCE-motivated engineered protections are designed to close.

### 1.4. Real-World Applicability

The detection framework evaluated in this paper is applicable to operational nuclear plants in several respects. The input data, 96 operational parameters including pressure, temperature, flow, and neutron flux, are standard measurements available in any PWR instrumented control system. The chi-squared detector requires only a period of normal-operation data for baseline estimation, which is available during commissioning, post-refueling startup, or steady-state operation. The threshold can be set to a target FAR that reflects operational tolerance. The per-accident detection analysis (Section 5.3) identifies which accident types are reliably detected and which require additional engineering protection, providing actionable guidance for safety-case development.

### 1.5. Contributions

The contributions of this work are fourfold:

1. A comparison of four lightweight anomaly detectors, chi-squared (Mahalanobis distance with Ledoit-Wolf shrinkage), Isolation Forest configured to match INL’s public AGN-201 deployment, per-feature z-score, and PCA reconstruction error, on the NPPAD dataset. All detectors are trained on normal operation only and evaluated under a random split of the normal data at a matched 1% target FAR (achieved FAR 0.98%), so comparisons reflect detection capability rather than threshold tuning. Chi-squared achieves AUC = 0.9989 (95% CI [0.9975, 0.9997]) and a 96.61% sample-level detection rate, outperforming Isolation Forest (AUC = 0.9762, 81.79% detection rate; DeLong $p=7.3\times 10^{-5}$), per-feature z-score (AUC = 0.9932; p=0.0016), and PCA reconstruction (AUC = 0.9903; p=0.031).
2. Identification of which accident types are detected (all 17 scenarios at the scenario level; 15 of 17 exceeding 95% sample-level detection; 14 of 17 at 100% sample-level detection) and which exhibit lower detection rates (load rejection at 70.6%, rod insertion at 92.5%, turbine trip at 97.1%), with physical explanation of the detection gaps and per-accident confidence intervals.
3. Application of the CCE Phase 4 principle to categorize NPPAD operational parameters by criticality, identifying which sensor pathways would require engineered protection beyond detection.
4. A synthetic false-data-injection stress test that operationalizes the threat model and demonstrates that the 96.61% physical-accident detection rate does not transfer to stealthy adversarial drift: at a drift magnitude of 0.5σ, chi-squared detection remains at 1.5% ± 0.7%, indistinguishable from its 0.98% FAR. This experiment bridges the gap between physical accident detection and the adversarial threat model, quantifying the CCE principle that engineered protections must complement detection.

The remainder of this paper is organized as follows. Section 2 introduces the N-RHES model. Section 4 describes the NPPAD dataset, the detection pipeline, and the detectors compared. Section 5 reports AUC, false alarm rate, per-accident detection rate, and comparison against published baselines. Section 5.9 discusses limitations, including class imbalance, the absence of adversarial scenarios in NPPAD, and the physical-accident-versus-cyberattack distinction. Section 6 concludes the paper.

## 2. System Modeling

N-RHES couple a nuclear reactor core with intermittent renewable generation [1] and, in many designs, industrial heat applications such as hydrogen production. The dynamic behavior of the nuclear subsystem under load-following conditions is governed by the interaction between neutron kinetics, thermal-hydraulic transport, and reactivity feedback. This section establishes the physical model that motivates the anomaly signatures observed in the NPPAD dataset and that frames the detection problem addressed in subsequent sections.

The model presented here is a representation implemented in Python 3.15. It is not a high-fidelity reactor simulator. The model serves three purposes: (1) to explain the physical origin of the operational parameters that constitute the NPPAD dataset, (2) to provide a reference for interpreting which accident signatures are detectable by distance-based methods and which are not, and (3) to define the bounded-drift attack model (Equation (10)) that is operationalized in the synthetic false-data-injection stress test of Section 5.7.

### 2.1. Point-Reactor Kinetics and Thermal Hydraulics

The core nuclear dynamics are represented by point-reactor kinetics coupled with lumped fuel and coolant temperatures. Point-reactor kinetics is the standard reduced-order model for transient analysis when spatial flux redistribution is not dominant, and it is the same model used in the PCTRAN simulator that generated the NPPAD dataset [31,35]. The governing equations are

(1) $$\begin{array}{cc} \dot{n}\left(t\right) & =\frac{\rho (t)-\beta }{\Lambda }n\left(t\right)+\sum_{i=1}^{6}\lambda_{i}C_{i}\left(t\right)+d_{n}\left(t\right), \end{array}$$

(2) $$\begin{array}{cc} {\dot{C}}_{i}\left(t\right) & =\frac{\beta_{i}}{\Lambda }n\left(t\right)-\lambda_{i}C_{i}\left(t\right),\;i=1,\ldots ,6, \end{array}$$

(3) $$\begin{array}{cc} {\dot{T}}_{f}\left(t\right) & =\frac{1}{M_{f}c_{f}}\left[P_{0}n\left(t\right)-U_{a}\left(T_{f}\left(t\right)-T_{c}\left(t\right)\right)\right], \end{array}$$

(4) $$\begin{array}{cc} {\dot{T}}_{c}\left(t\right) & =\frac{1}{M_{c}c_{c}}\left[U_{a}\left(T_{f}\left(t\right)-T_{c}\left(t\right)\right)-{\dot{m}}_{cool}\left(t\right)c_{c}\left(T_{c}\left(t\right)-T_{in}\right)\right], \end{array}$$

where n(t) is the relative neutron population density normalized to nominal full power, $C_{i}\left(t\right)$ represents the concentration of the *i*-th delayed neutron precursor group, $T_{f}\left(t\right)$ and $T_{c}\left(t\right)$ denote lumped fuel and coolant temperatures, $T_{in}$ is the coolant inlet temperature, and ρ(t) is the total reactivity, which combines external control rod action and temperature feedback. The parameters β and $\beta_{i}$ are the total and group-wise delayed neutron fractions, Λ is the prompt neutron generation time, and $\lambda_{i}$ are the precursor decay constants. The term $d_{n}\left(t\right)$ represents unmodeled disturbances or, in a cybersecurity context, adversarial manipulation of the neutron detector signal.

The thermal-hydraulic equations represent a two-node lumped model: fuel-to-coolant heat transfer is governed by the overall heat transfer coefficient $U_{a}$, and the coolant loop removes heat at a mass flow rate ${\dot{m}}_{cool}\left(t\right)$. The heat capacities $M_{f}c_{f}$ and $M_{c}c_{c}$ are lumped quantities. This level of fidelity is sufficient to reproduce the qualitative transient behavior of a pressurized water reactor under load-following, which is the regime relevant to N-RHES operation.

The reactivity ρ(t) is decomposed as

(5) $$\rho \left(t\right)=\rho_{\mathrm{ext}}\left(t\right)+\alpha_{f}\left(T_{f}\left(t\right)-T_{f,\mathrm{ref}}\right)+\alpha_{c}\left(T_{c}\left(t\right)-T_{c,\mathrm{ref}}\right),$$

where $\rho_{\mathrm{ext}}\left(t\right)$ is the externally imposed reactivity from control rod motion or boron injection, and $\alpha_{f}$ and $\alpha_{c}$ are the fuel and coolant temperature coefficients of reactivity. Both coefficients are negative in a typical light-water reactor, providing the self-stabilizing feedback that limits power excursions.

### 2.2. Coupled Intermittency and Xenon Constraints

In an N-RHES configuration, the nuclear subsystem is coupled to a volatile renewable generation term $P_{ren}\left(t\right)$, which forces the reactor into dynamic load, following the coolant mass flow rate and how the reactor power setpoint respond to the renewable mismatch, producing the transient conditions under which accident scenarios such as loss of flow, load rejection, or steam generator tube rupture become operationally relevant. These are precisely the scenarios represented in the NPPAD dataset [31].

Safety bounds are defined by the admissible safe set C:

(6) $$C=\left\{x\in R^{n}|T_{f,\min}\leq T_{f}\left(t\right)\leq T_{f,\max},\;\left|X_{e}\left(t\right)\right|\leq X_{e,\mathrm{limit}}\right\},$$

where $X_{e}\left(t\right)$ represents the xenon-135 concentration. Xenon-135 is a fission product with a large thermal neutron absorption cross-section [36], and its dynamics introduce a slow reactivity feedback that constrains load-following maneuvers. The xenon concentration evolves according to

(7) $$\begin{array}{cc} \dot{I}\left(t\right) & =\gamma_{I}\Sigma_{f}\phi \left(t\right)-\lambda_{I}I\left(t\right), \end{array}$$

(8) $$\begin{array}{cc} {\dot{X}}_{e}\left(t\right) & =\gamma_{Xe}\Sigma_{f}\phi \left(t\right)+\lambda_{I}I\left(t\right)-\lambda_{Xe}X_{e}\left(t\right)-\sigma_{Xe}\phi \left(t\right)X_{e}\left(t\right), \end{array}$$

where I(t) is the iodine-135 precursor concentration, ϕ(t) is the neutron flux (proportional to n(t)), $\gamma_{I}$ and $\gamma_{Xe}$ are the fission yields of iodine and xenon, $\lambda_{I}$ and $\lambda_{Xe}$ are their decay constants, and $\sigma_{Xe}$ is the xenon microscopic absorption cross-section. The xenon constraint in Equation (6) captures the operational requirement that load-following maneuvers must not drive the reactor into a xenon-induced spatial power oscillation or a reactivity deficit that cannot be recovered by control rod action.

### 2.3. Relation to the NPPAD Dataset

The NPPAD dataset was generated using PCTRAN, a simulator whose reactor dynamics module implements the same point-reactor kinetics Equations (1) and (2) and whose thermal-hydraulic module is based on first-principles mass and energy balance [31]. The 96 operational parameters recorded in NPPAD, including reactor pressure, coolant temperatures, mass flow rates, pressurizer level, and steam generator parameters, are direct observables of the state variables in (1)–(4) and their associated subsystems. This correspondence is important for interpreting the detection results in Section 5: the anomaly signatures that distinguish normal operation from accident conditions are physical deviations in these state variables, not arbitrary statistical artifacts.

This physical grounding directly informs the detector design choices. The chi-squared detector’s covariance structure reflects the correlated deviations that emerge from the coupled kinetics and thermal-hydraulics, a loss-of-coolant accident, for example, produces simultaneous changes in pressure, temperature, and flow that are jointly anomalous even if no single parameter is extreme. The per-accident analysis in Section 5.3 shows that this correlated structure is precisely what allows chi-squared to achieve 96.61% sample-level detection. Conversely, the scenarios with lower detection rates, load rejection and rod insertion, are compensated transients in which the control system (governed by the same reactivity feedback equations) partially absorbs the disturbance, keeping the joint parameter deviations within the normal envelope for an extended period. The physical model thus explains both the detector’s success and its specific failure modes.

At the same time, it is essential to state clearly what this model does not establish. The equations above describe physical accident transients, not adversarial cyber manipulations. A false-data-injection attack on a sensor would alter the measured value of a state variable while leaving the physical state itself unchanged; the resulting signature in the measurement space depends on the attacker’s strategy and may differ substantially from the signatures of physical accidents. The NPPAD dataset does not contain such adversarial manipulations. The detection benchmark reported in this paper therefore characterizes the detectability of physical accident signatures, and the extension to adversarial scenarios is addressed separately through the synthetic false-data-injection stress test of Section 5.7, which is explicitly labeled as synthetic and does not claim to represent real cyberattacks.

### 2.4. Modeling Assumptions

The model rests on the following assumptions:

**Assumption 1.**  *Fuel and coolant are each represented by a single temperature node. This neglects spatial temperature gradients and is appropriate for qualitative transient analysis but not for localized phenomena such as hot-channel effects.*

**Assumption 2.**  *The standard six-group representation is used, consistent with the PCTRAN implementation [35].*

**Assumption 3.**  *The xenon model in Equations (7) and (8) neglects spatial xenon oscillation modes, which can become important under aggressive load-following.*

**Assumption 4.**  *The renewable generation term $P_{ren}\left(t\right)$ is treated as an exogenous input rather than as a coupled dynamical system. This simplification is acceptable for the present purpose, which is to motivate the physical scenarios represented in NPPAD rather than to simulate an integrated N-RHES plant.*

These assumptions are consistent with the level of fidelity required to interpret NPPAD accident signatures. They are not sufficient for safety analysis or for design certification, and they are not presented as such.

## 3. Threat Model

The detection benchmark presented in this paper is motivated by a specific adversarial scenario that we articulate using the CCE framework. It is essential to distinguish two layers of this section: (1) the conceptual threat model, which defines the High-Consequence Event (HCE) and the attack pathway that motivates the detection problem, and (2) the empirical evaluation, which is conducted on the NPPAD dataset of physical accident transients. The NPPAD dataset does not contain adversarial manipulations, and the empirical results in Section 5 therefore characterize detection of physical anomalies, not detection of cyberattacks. The connection between the threat model and the empirical evaluation is established through the synthetic false-data-injection stress test of Section 5.7, which operationalizes Equation (10) with a bounded random-walk injection into held-out normal data. We state this distinction explicitly to prevent misreading of the results.

### 3.1. Consequence-Driven Targeting

Following Consequence-driven Targeting [8], we adopt an attacker’s perspective and ask: how would an adversary manipulate N-RHES digital assets to achieve an HCE? The CCE methodology explicitly prioritizes reasoning from consequences backward to attack pathways, rather than reasoning forward from known vulnerabilities. This inversion is what makes CCE distinct from conventional threat modeling, it begins with the unacceptable outcome and asks what would have to be true for that outcome to occur.

We define the HCE for the N-RHES context as follows:

**Definition 1**  **(HCE).** *Sustained violation of the fuel temperature safety limit $T_{f,\max}$ during a renewable intermittency transient, resulting in fuel damage or an automatic scram that disables grid-balancing capability.*

This HCE is chosen because it couples a physical safety consequence (fuel damage) with an operational consequence (loss of grid-balancing function), and because it is the kind of event that a consequence-driven analysis is designed to prevent. The CCE methodology’s central principle is that such events should be prevented through engineered protections rather than through detection alone, because detection can fail in adversarial conditions where the attacker actively shapes the signature to evade it.

### 3.2. Attack Pathway (Stealthy Sensor Calibration Drift)

The attack pathway we consider is a cumulative sensor calibration drift, introduced during a pre-positioning phase and remaining below conventional residual thresholds during steady-state operation. The adversary’s objective is to corrupt the measurement of fuel or coolant temperature such that the control system’s response to a subsequent renewable intermittency transient is incorrect, driving the plant toward the HCE.

Formally, let $x\left(t\right)\in R^{n}$ denote the true plant state, and let *C* denote the measurement matrix. The corrupted measurement vector available to the monitoring and control system is

(9) y(t)=Cx(t)+ϵ(t)+w(t),

where w(t) is zero-mean sensor noise and ϵ(t) is the adversarial drift. The drift is modeled as a slowly varying state governed by

(10) $$\dot{\epsilon }\left(t\right)=A_{adv}\epsilon \left(t\right)+\eta \left(t\right),\;∥\dot{\epsilon }\left(t\right)∥\leq \nu ,$$

where $A_{adv}$ is a stable adversarial dynamics matrix and η(t) is a bounded injection signal. The critical property is that ν→0 during steady-state operation, so that standard residual-based detectors of the form $∥y-C\hat{x}∥\leq \tau$ remain un-tripped. The drift is designed to remain invisible until a triggering condition occurs. The synthetic FDI experiment of Section 5.7 implements a simplified bounded random walk that preserves the defining property $∥\dot{\epsilon }∥\leq \nu$; the generalization to state-dependent adversarial dynamics is identified as future work.

The triggering condition is a sharp drop in renewable generation $P_{ren}\left(t\right)$. When this occurs, the control system must respond to a rapidly changing thermal-hydraulic state, and the corrupted measurement causes it to respond incorrectly. The result is a thermal-hydraulic shock that the plant’s safety systems may not be able to absorb before the fuel temperature exceeds its limit. This is the mechanism by which a pre-positioned, low-magnitude corruption achieves a high-consequence outcome.

This framing is consistent with CCE logic: we are not discovering a new attack; we are applying the CCE framework to a known attack class within the N-RHES context. The CCE methodology’s value is that it forces the analyst to reason about the consequence first and the attack mechanism second, which is what allows the analysis to identify which sensors are critical choke points and which are not.

Finally, we note that the threat model is evaluated empirically in the synthetic FDI experiment (Section 5.7), which demonstrates that at a stealthy drift magnitude of 0.5σ, the chi-squared detector’s detection rate remains at 1.5% ± 0.7%, indistinguishable from its 0.98% false alarm rate. This quantifies the gap between physical accident detection and adversarial manipulation detection, supporting the CCE principle that engineered protections must complement detection.

### 3.3. Relation to the Empirical Evaluation and CCE Implications

The threat model above defines the conceptual problem. The primary empirical evaluation is conducted on the NPPAD dataset [31], which contains physical accident transients rather than adversarial sensor manipulations. The results in Section 5 therefore establish the relative performance of chi-squared, Isolation Forest, and other detectors on physical accident signatures. The synthetic FDI experiment in Section 5.7 complements this benchmark by providing a controlled, synthetic test of adversarial behavior under the attack pathway described above.

This distinction is a characterization of what can be validated on publicly available security data. The NPPAD benchmark provides the strongest publicly available evidence on detector behavior in the physical regime, while the synthetic FDI experiment provides the complementary evidence on adversarial behavior without requiring a labeled cyberattack dataset or hardware-in-the-loop testbed, both of which remain outside the scope of this work.

A consequence of this analysis is that detection alone is not sufficient to prevent the HCE. The synthetic FDI experiment empirically demonstrates this: even a detector that performs well on physical accidents, such as the chi-squared detector evaluated in Section 5, fails against a stealthy drift designed to remain within the normal operating envelope. This is precisely the situation that CCE is designed to address, and it motivates the principle that engineered protections should be prioritized over detection-based defenses.

In the N-RHES context, the CCE-informed mitigation would be to engineer out the dependence on potentially corrupted digital signals for safety-critical functions. For example, a redundant analog trip path that does not rely on the digital temperature measurement could provide a safety backstop that is immune to sensor calibration drift. This principle, that independent hardware-level analog safeguards can enforce safety constraints even when the digital control layer is compromised or corrupted, is central to the Guarded Swarms framework of Borghoff et al. [37], which demonstrates that analog guard channels operating outside the software stack can unilaterally veto unsafe commands, independently of software state in cyber-physical systems. While the synthetic FDI experiment does not evaluate any specific engineered protection, it establishes the quantitative motivation for pursuing them. This framework provides an example of how such protections can be realized, though evaluating a specific analog trip path in the NPPAD context is the subject of future work. The detection benchmark reported in this paper is therefore a necessary but not sufficient component of a CCE-compliant defense; the engineered protections that complement it are the subject of future work.

## 4. Detection Pipeline and CCE-Informed Mitigation

This section describes the detection pipeline evaluated in this paper. The pipeline has two components: (1) a set of anomaly detectors applied to NPPAD operational parameters, and (2) a CCE-informed mitigation analysis that identifies which sensor pathways are critical enough to warrant engineered protection. The detectors are evaluated comparatively, with chi-squared (Mahalanobis distance) as the primary detector and Isolation Forest (IFML) as the reference baseline. Two additional baselines, per-feature z-score and PCA reconstruction, are included for completeness.

### 4.1. Anomaly Detection Framework

We frame anomaly detection as a semi-supervised problem. The detector is trained on normal operation data only, and it must identify deviations from that baseline at test time without ever having seen an accident scenario during training. This is the operational regime in a nuclear plant: accidents are rare, labeled accident data is scarce, and a detector that requires labeled anomalies for training is impractical.

Formally, let $X_{\mathrm{train}}\in R^{n\times d}$ be a matrix of *n* normal-operation samples with *d* features. The detector produces a scoring function $s:R^{d}\to R$ and a threshold τ such that a new sample *x* is flagged as anomalous when s(x)>τ. The threshold is calibrated on training data to achieve a target FAR, which we set to 1% throughout this paper to reflect the operational tolerance for false alarms in a nuclear control room.

Four detectors are compared, described in Section 4.2, Section 4.3, Section 4.4 and Section 4.5. All are evaluated at the same FAR target, which ensures that comparisons reflect detection capability rather than threshold calibration.

### 4.2. Chi-Squared (Mahalanobis) Detection

The chi-squared detector assumes that normal-operation samples follow a multivariate Gaussian distribution with mean $\mu \in R^{d}$ and covariance $\Sigma \in R^{d\times d}$. Under this assumption, the squared Mahalanobis distance

(11) $$D^{2}\left(x\right)={\left(x-\mu \right)}^{⊤}\Sigma^{-1}\left(x-\mu \right),$$

follows a chi-squared distribution with *d* degrees of freedom when μ and Σ are known. In practice, the parameters are estimated from a finite training sample and shrinkage is applied, so the theoretical chi-squared quantile is only approximate. We therefore calibrate the threshold empirically from training data, τ is the (1−α) quantile of the training Mahalanobis distances, with α the target FAR. This avoids reliance on the asymptotic distribution.

The mean and covariance are estimated from training data. Because the number of training samples (302) is small relative to the number of features (96), the sample covariance matrix is ill-conditioned. When the sample size *n* and the feature dimension *p* are of comparable order, the sample covariance matrix is known to perform poorly and may not even be invertible. To address this, we use the linear Ledoit-Wolf shrinkage estimator [26], which regularizes the covariance toward a scaled identity matrix. The shrinkage intensity is selected automatically by minimizing the expected squared Frobenius norm between the estimated and true covariance [26]. This produces a well-conditioned Σ and a numerically stable $\Sigma^{-1}$, a property that holds even when the raw sample covariance matrix is ill-conditioned [26]. Section 5.6 reports a sensitivity analysis across shrinkage intensities, showing that the AUC varies by less than 0.001 over the range tested.

The Mahalanobis distance has a useful interpretation in the nuclear context, it measures how many standard deviations a sample lies from the normal operating envelope, accounting for correlations between parameters. A steam generator tube rupture, for example, produces correlated deviations in pressure, temperature, and flow that are jointly anomalous even if no single parameter is extreme. The Mahalanobis distance captures this joint deviation naturally, which is why it is expected to perform well on physically structured accident signatures.

### 4.3. Isolation Forest Detection

The Isolation Forest (IFML) algorithm [38] is a non-parametric anomaly detector that recursively partitions the data space with random splits. Anomalies are points that are isolated after relatively few splits, yielding shorter average path lengths across an ensemble of isolation trees. The theoretical anomaly score is

(12) $$s\left(x,\psi \right)=2^{-\frac{E[h(x)]}{c(\psi )}},$$

where E[h(x)] is the average path length across trees, ψ is the subsample size, and c(ψ) is the expected path length of an unsuccessful search in a binary search tree. In this formulation, higher s(x) indicates a more anomalous point: a shorter path length E[h(x)] produces a smaller exponent magnitude, giving a value of *s* closer to 1 (the maximum of the score). A normal point, with a longer average path length, produces an *s* closer to 0.

The scikit-learn implementation uses decision_function() returns s(x,ψ)−0.5, so that the values lie in [−0.5,0.5] with lower (more negative) values indicating more anomalous points. To align with our threshold convention (where larger scores indicate greater anomaly, consistent with the other detectors), we negate the decision_function() output: $s_{\mathrm{impl}}\left(x\right)=-\mathrm{decision}\_\mathrm{function}\left(x\right)$. This preserves the ordering, larger $s_{\mathrm{impl}}$ means more anomalous, while ensuring a consistent threshold direction across all detectors. The threshold τ is then the 99th percentile of $s_{\mathrm{impl}}$ on the training set, and a test sample is flagged as anomalous when $s_{\mathrm{impl}}\left(x\right)>\tau$, exactly as for the other detectors.

We implement IFML using scikit-learn [39] with the same configuration reported in [10]: contamination=‘auto’, n_estimators=100, random_state=42. The contamination parameter controls the assumed proportion of anomalies in the training data. It affects only the model’s predict() decision boundary, not the continuous decision_function() score on which our threshold is based. We therefore report a single IFML configuration.

IFML is the reference baseline, the choice is justified on a peer-reviewed nuclear benchmark. The comparison is not a claim about INL’s deployment, which operates on different data and with different operational constraints.

### 4.4. Per-Feature Z-Score Baseline

The per-feature z-score detector is a univariate baseline that flags a sample when any single feature deviates from its normal mean by more than a threshold number of standard deviations:

(13) $$s_{z}\left(x\right)=\max_{j}\left|\frac{x_{j}-\mu_{j}}{\sigma_{j}}\right|,$$

where $\mu_{j}$ and $\sigma_{j}$ are the mean and standard deviation of feature *j* in the training set. This detector ignores correlations between features. It is included because it represents the simplest possible anomaly detection strategy and provides a lower bound on what any reasonable detector should achieve.

### 4.5. PCA Reconstruction Error Baseline

Principal Component Analysis (PCA) [40] projects the data onto the top-*k* principal components of the training set and measures the reconstruction error:

(14) $$s_{\mathrm{PCA}}\left(x\right)={∥x-V_{k}V_{k}^{⊤}\left(x-\mu \right)-\mu ∥}^{2},$$

where $V_{k}$ contains the top-*k* principal component directions. This detector captures linear subspace structure: normal samples lie near the *k*-dimensional principal subspace, and anomalies deviate from it. We use k=10 components, which captures the dominant modes of variation in the training data while retaining enough residual structure to distinguish anomalies.

The PCA detector is included because it is a standard baseline in the anomaly detection literature and because it provides a linear alternative to the full-covariance Mahalanobis distance. If PCA reconstruction and chi-squared perform similarly, the additional structure captured by the full covariance is not contributing to detection.

### 4.6. CCE-Informed Mitigation Analysis

The detection pipeline described above addresses the question: can we identify anomalies in operational data? It does not address the deeper question that CCE is designed to answer: what happens if the detector fails? The CCE methodology’s principle is that engineered protections should be prioritized over detection-based defenses because detection can be defeated by adversaries who shape their attack to evade it [8].

We apply this principle by identifying the sensor pathways that, if corrupted, would directly enable the HCE defined in Section 3. For each pathway, we ask whether an engineered protection, such as a redundant analog trip circuit, a hardware interlock, or a physically independent measurement, could provide a safety backstop that does not depend on the digital signal. The pathways that cannot be backed up in this way are flagged as critical choke points requiring additional design attention.

This analysis is qualitative rather than quantitative. It is based on the structure of the NPPAD operational parameters and on the CCE framework’s prioritization logic, not on a formal fault tree or probabilistic risk assessment. The synthetic false-data-injection stress test of Section 5.7 provides a complementary quantitative assessment: it demonstrates empirically that the detector’s performance on physical accidents does not transfer to stealthy adversarial drift, which is precisely the gap that engineered protections are designed to close. A full CCE analysis would require plant-specific design information that is not available in a public dataset.

## 5. Experimental Evaluation and Results

### 5.1. Data Preparation and Evaluation Protocol

The NPPAD dataset provides 302 normal-operation samples in a single file (1.csv) and 17 accident directories containing thousands of samples each. We use the CSV-format files in Operation_csv_data, retain the 96 operational parameters common to all directories, and exclude the TIME column from the feature set. No normal samples contain missing or infinite values in the retained features, we nonetheless replace any such values with zero as a defensive measure. Accident samples are drawn from up to 20 CSV files per directory, yielding 110,369 accident samples across 17 scenarios.

#### 5.1.1. Evaluation Splits

Since NPPAD provides only one normal-operation file, the choice of how to split the 302 normal samples is consequential. We adopt a random split as the primary evaluation protocol: 200 samples are used for training and the remaining 102 are held out as an independent normal test set. We additionally compute a temporal split (first 200 for training, last 102 for testing) and report it as a diagnostic, but not as a primary result.

**Figure 1:**
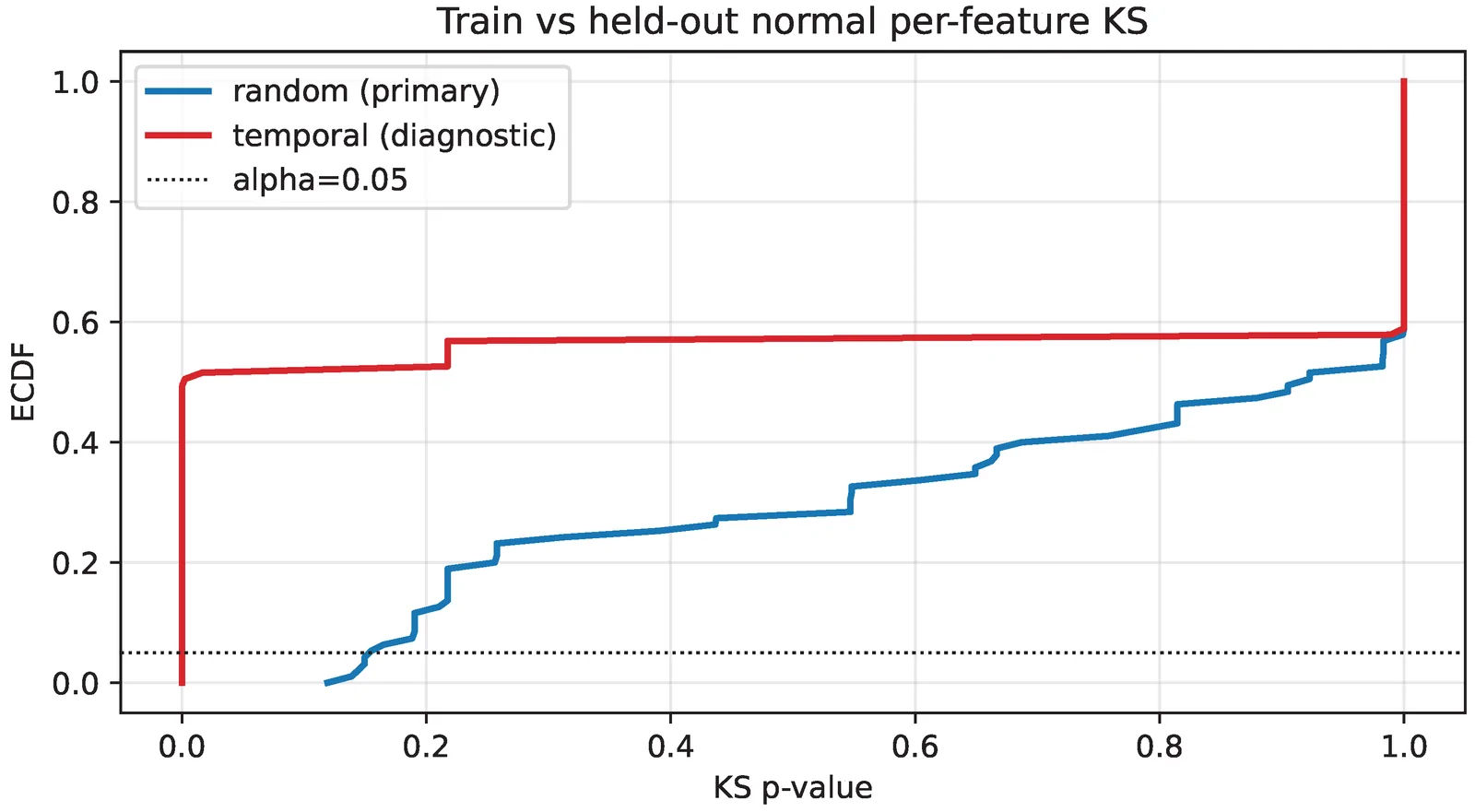
Empirical CDF of per-feature Kolmogorov-Smirnov (KS) *p*-values comparing training and held-out normal samples. Under the random split (blue), all *p*-values exceed 0.10, resulting in 0.0% of features rejecting exchangeability at α=0.05 and confirming that a random split yields a valid false alarm estimate. Under the temporal split (red), 52.1% of features have *p*-values near zero (p<0.05), demonstrating that the NPPAD normal-condition record is non-stationary and that a temporal holdout cannot support a valid FAR estimate.

The reason for preferring the random split is empirical. Figure 1 reports the empirical CDF of per-feature Kolmogorov-Smirnov (KS) *p*-values comparing training and held-out normal samples. Under the random split, the *p*-values are uniformly distributed, with 0.0% of features rejecting exchangeability at α=0.05: train and test samples are statistically indistinguishable, so the held-out normal set provides a valid estimate of false alarm behavior. Under the temporal split, 52.1% of features reject exchangeability, and the achieved FAR rises to over 30%. A temporal holdout from a single normal record is therefore not a valid basis for false alarm estimation in this dataset.

We emphasize that the random split is chosen for estimation of the false alarm rate, not as a claim that the data are temporally uncorrelated. Consecutive measurements within the same operating record are temporally correlated at the 10 s sampling interval, and this correlation affects both the normal and accident sets. We address the correlation in two ways. First, all confidence intervals for AUC use a block bootstrap that resamples accident files rather than individual samples (Section 5.1.3), so the within-file correlation structure is preserved. Second, the achieved FAR of 0.98% under the random split is an estimate of the false alarm probability under exchangeable sampling; the temporal split’s FAR exceeding 30% primarily reflects the non-stationarity of the single 302-sample normal record (52.1% of features reject exchangeability) rather than a defect in the detector. A fully independent normal test set from a different operating period would be required to establish the field FAR (Section 5.10).

#### 5.1.2. Threshold Calibration

All detectors are calibrated to a 1% target FAR on the training data only. The threshold for each detector is the 99th percentile of training scores: for chi-squared and PCA, the 99th percentile of the training anomaly score; for Isolation Forest, the 99th percentile of the training decision function; for per-feature z-score, the 99th percentile of the maximum absolute training z-score. Thresholds are frozen before any test evaluation and are never recomputed on test data, normal or accident.

#### 5.1.3. Metrics and Statistical Analysis

Because the test set is overwhelmingly composed of accident samples (99.91%; 110,369 accident vs. 102 normal), precision-based metrics are saturated near 1.0 and are not informative for ranking detectors. We therefore report four metrics: (i) AUC, the threshold-independent area under the ROC curve, with a 95% block-bootstrap confidence interval resampling accident files to respect within-file temporal correlation; (ii) achieved FAR, the fraction of the 102 held-out normal samples flagged at the frozen threshold, with a Clopper-Pearson 95% confidence interval; (iii) sample-level detection rate, the fraction of accident samples flagged; and (iv) scenario-level detection, the fraction of accident scenarios for which at least one sample is flagged. The distinction between sample-level and scenario-level criteria is important: all 17 scenarios are detected at the scenario level, while only a subset achieve high sample-level rates (Section 5.3).

An exact DeLong test is used to assess the significance of AUC differences between chi-squared and each baseline. Because the DeLong test assumes independent observations and the accident samples are temporally correlated within files, we additionally report a cluster-aware bootstrap test that resamples accident files (blocks) and normal samples independently across 1000 resamples. The cluster-aware test confirms the DeLong conclusions: chi-squared vs. Isolation Forest ΔAUC=0.0227, $p<10^{-3}$, 95% CI [0.0116,0.0362]; vs. per-feature z-score ΔAUC=0.0057, $p<10^{-3}$, 95% CI [0.0022,0.0104]; vs. PCA ΔAUC=0.0086, p=0.030, 95% CI [0.0005,0.0177]. Both the DeLong and cluster-aware results are reported in Table 1.

#### 5.1.4. Reproducibility

All experiments use np.random.seed(42) and random_state=42 for scikit-learn estimators. Chi-squared, per-feature z-score, and PCA are deterministic given the training data; randomness enters only through Isolation Forest’s split selection, fixed by the seed, and through the bootstrap. All reported numbers are generated by a single execution of the evaluation script described in Section 4. The synthetic FDI experiment is the only component with an additional seed; it uses $N_{\mathrm{rep}}=10$ realizations per drift magnitude, with seeds FDI_SEED+r for r=0,…,9, where FDI_SEED=42 is the base seed defined in the evaluation script. The reported FDI detection rates are the mean over these 10 realizations. The parameter settings are those specified in Section 4.2, Section 4.3, Section 4.4 and Section 4.5.

### 5.2. Detector Comparison

Table 1 summarizes performance on the held-out test set. Chi-squared achieves the highest AUC (0.9989, 95% CI [0.9975,0.9997]) and the highest sample-level detection rate (96.61%) at an achieved FAR of 0.98% (95% CI [0.02%,5.34%]). Isolation Forest achieves AUC 0.9762 and detection rate 81.79%. Per-feature z-score achieves AUC 0.9932 and detection rate 89.53%. PCA reconstruction achieves AUC 0.9903 and detection rate 89.57%. The DeLong test rejects equality of AUCs for chi-squared vs. Isolation Forest ($p=7.3\times 10^{-5}$), vs. per-feature z-score (p=0.0016), and vs. PCA (p=0.031), establishing that the chi-squared advantage is statistically significant at conventional levels. The cluster-aware bootstrap test, which respects within-file temporal correlation, confirms these conclusions: ΔAUC=0.0227 vs. Isolation Forest ($p<10^{-3}$), 0.0057 vs. per-feature z-score ($p<10^{-3}$), and 0.0086 vs. PCA (p=0.030).

We note explicitly that the Clopper-Pearson interval for FAR at 102 held-out normal samples is wide ([0.02%,5.34%]). The 102-sample normal set cannot resolve a 1% from a 3% true false alarm rate, and we do not use FAR to rank detectors. Detector ranking rests on AUC and detection rate, which are computed over the 110,369 accident samples and are far better powered. This is a limitation of NPPAD, not of the detectors, and is discussed further in Section 5.9.

Because the achieved FARs differ across detectors at the same 1% target (chi-squared 0.98%, per-feature z-score 0.98%, Isolation Forest 2.94%, PCA 4.90%), we also report the sample-level detection rate at a common achieved FAR of 2.94% (the Isolation Forest operating point). At this operating point, chi-squared achieves 98.72% detection, per-feature z-score 91.19%, PCA 81.34%, and Isolation Forest 81.21%. The chi-squared advantage persists at a matched operating point, not only at its own achieved FAR. The Clopper-Pearson interval widths for the achieved FARs are 0.053 (chi-squared and z-score), 0.078 (Isolation Forest), and 0.095 (PCA); these widths quantify the uncertainty arising from the limited 102-sample normal set, and the overlap of the chi-squared and z-score intervals confirms that their comparison is at the same operating point.

The differences in achieved FAR across detectors are not evidence of miscalibration; they arise from sampling variance on the 102 held-out normal samples. Each detector is calibrated to a 1% target FAR on the 200 training samples (approximately two expected false alarms in training), and the achieved FAR on the 102 held-out samples is a binomial draw whose 95% interval spans [0.02%,5.34%]. The observed range of 0.98–4.90% is therefore within the sampling noise of the calibration. Importantly, the FAR differences do not confound the detector ranking in favor of chi-squared: chi-squared has the lowest achieved FAR (0.98%), i.e., the most conservative operating point, which would ordinarily reduce rather than increase its detection rate. Despite this, chi-squared achieves the highest sample-level detection rate (96.61%) and the highest TPR at the common 2.94% FAR (98.72%). The ranking is therefore robust to the FAR differences, and the matched-FAR comparison reinforces rather than reverses the conclusion.

### 5.3. Per-Accident Detection Analysis

**Figure 2:**
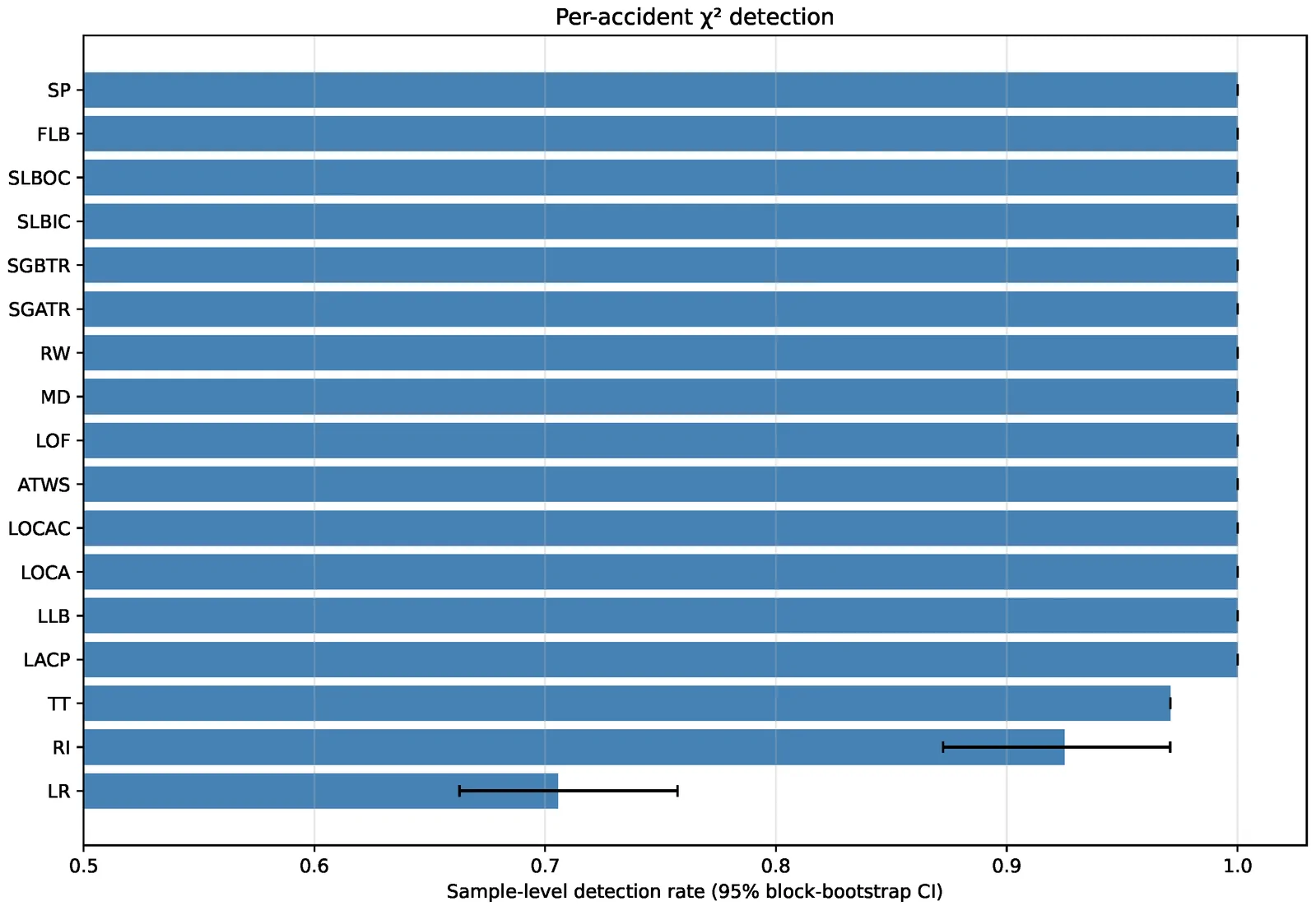
Sample-level detection rate for each of the 17 accident scenarios using the chi-squared detector at the frozen 1% false alarm threshold. Error bars indicate 95% block-bootstrap confidence intervals (resampling at the accident-file level). Out of 17 scenarios, 14 achieve 100% detection; 15 exceed 95%. The 2 exceptions, load rejection (70.6%), and rod insertion (92.5%), share a physical characteristic: they are compensated transients in which the control system partially absorbs the initial disturbance, keeping a subset of post-transient samples within the normal operating envelope.

Figure 2 reports the sample-level detection rate of the chi-squared detector for each of the 17 accident types, with 95% block-bootstrap confidence intervals. All 17 scenarios are detected at the scenario level, at least one sample per file is flagged in every scenario. At the sample level, 14 of 17 scenarios achieve 100% detection and 15 of 17 exceed 95%. Two types fall below the 95% threshold, LR (load rejection) at 70.6%[66.3%,75.7%], and RI (rod insertion) at 92.5%[87.2%,97.1%]. TT (turbine trip) achieves 97.1%, above the threshold, but is the third-lowest-detection scenario. Table 2 reports the full per-accident breakdown, including the number of files per scenario and the file-macro-averaged detection rates.

**Figure 3:**
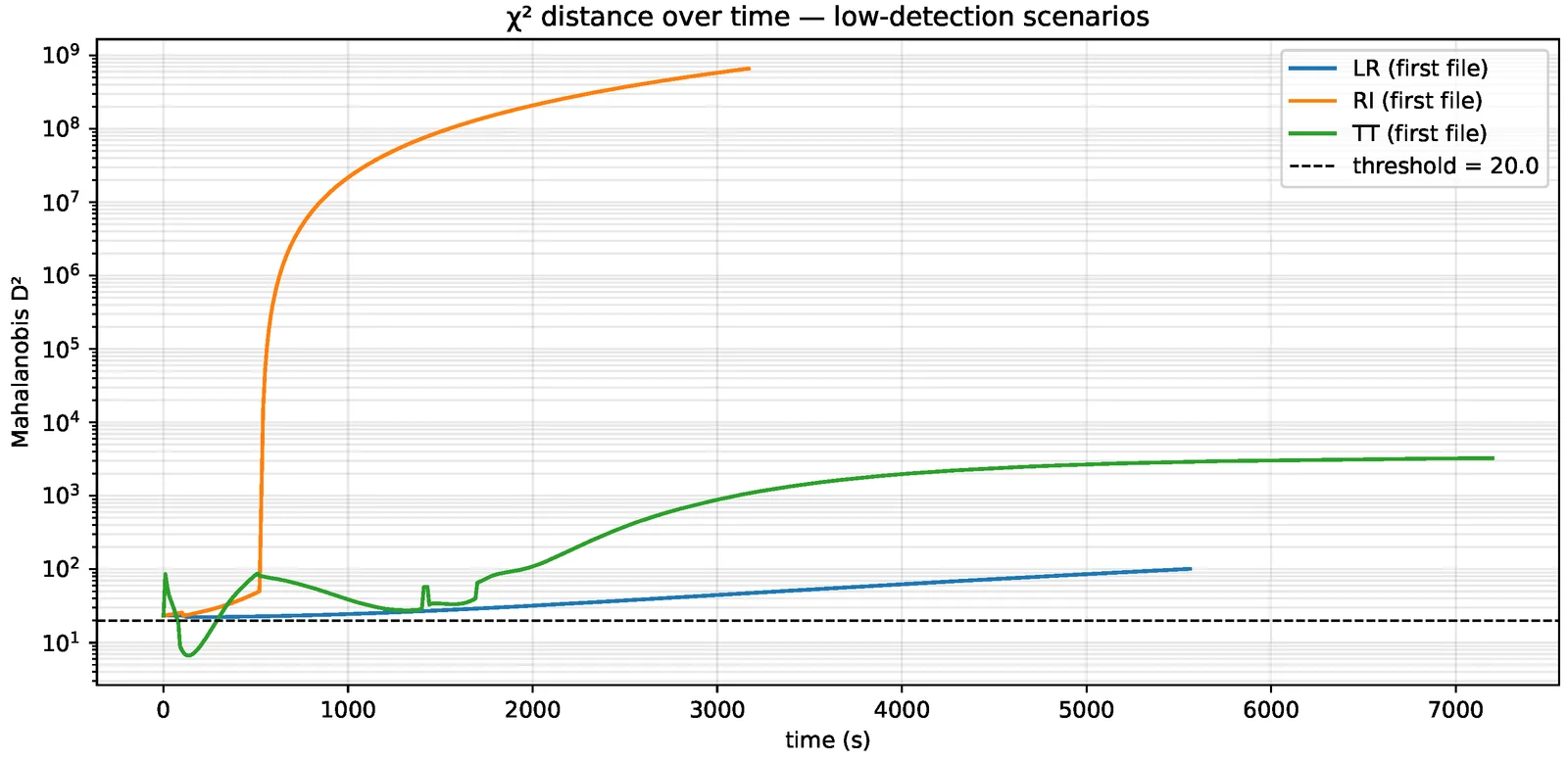
Squared Mahalanobis distance ($D^{2}$, log scale) trajectory over time for the three lowest-detection accident scenarios: load rejection (LR), rod insertion (RI), and turbine trip (TT). The dashed horizontal line is the frozen detection threshold (τ=20.0). TT exhibits an initial spike at fault initiation, dips briefly below the threshold, and crosses around t≈350s; RI stays near normal levels initially before experiencing a steep exponential rise past the threshold at approximately t≈500s; LR begins near the threshold boundary at t=0 and gradually drifts upward without dipping below it. These delayed or gradual threshold crossings reflect the physical nature of these compensated transients: the control system partially absorbs the initial disturbance, keeping joint parameter deviations within or close to the normal operating envelope for an extended duration.

The three lowest-detection types (LR, RI, and TT) share a physical characteristic: their transient signatures partially overlap with the normal operating envelope in the 96-dimensional feature space. Load rejection, for example, produces a brief power reduction and a temperature excursion that the control system partially compensates within a few seconds, so the post-transient state returns close to the pre-transient state. Rod insertion and turbine trip produce similar partial-overlap signatures. Figure 3 shows the Mahalanobis distance over time for the first file of each of these three scenarios. The trajectories confirm that the difficulty is not a failure to detect the transient but a delay: LR begins near the threshold boundary at t=0 and gradually drifts upward, crossing fully at approximately 4000 s; RI crosses at approximately 500 s but only after a large spike that yields a small fraction of flagged samples overall; and TT exhibits an initial spike at fault initiation, dips briefly below the threshold, and crosses at approximately 350 s. The chi-squared detector is expected to have difficulty with these signatures precisely because the Mahalanobis distance remains close to the threshold for a subset of samples, even though the events are physically distinct.

This per-accident analysis is the kind of diagnostic information that a CCE-informed operational deployment would use to prioritize engineering mitigations. For the 15 accident types with sample-level detection above 95%, detection may serve as a useful first layer, but high detection rates alone do not establish acceptable detection latency, operational reliability, or safety performance, those properties are not characterized by this simulation-based evaluation. For LR and RI in particular, the detection gap motivates the CCE principle of engineering protections that do not depend on the detector succeeding in every case.

We note one interpretation caveat. Because NPPAD accident files begin at fault initiation, the first post-trigger sample already exceeds the threshold in most scenarios, and the median time-to-detection is 0 s in the sampled files. The reported AUCs and detection rates therefore characterize separability of accident states from normal operation, not end-to-end onset-detection latency. Real deployment latency would depend on scan rate, filtering, and persistence logic not modeled here, and is discussed further in Section 5.9.

### 5.4. ROC and Score Distribution Analysis

**Figure 4:**
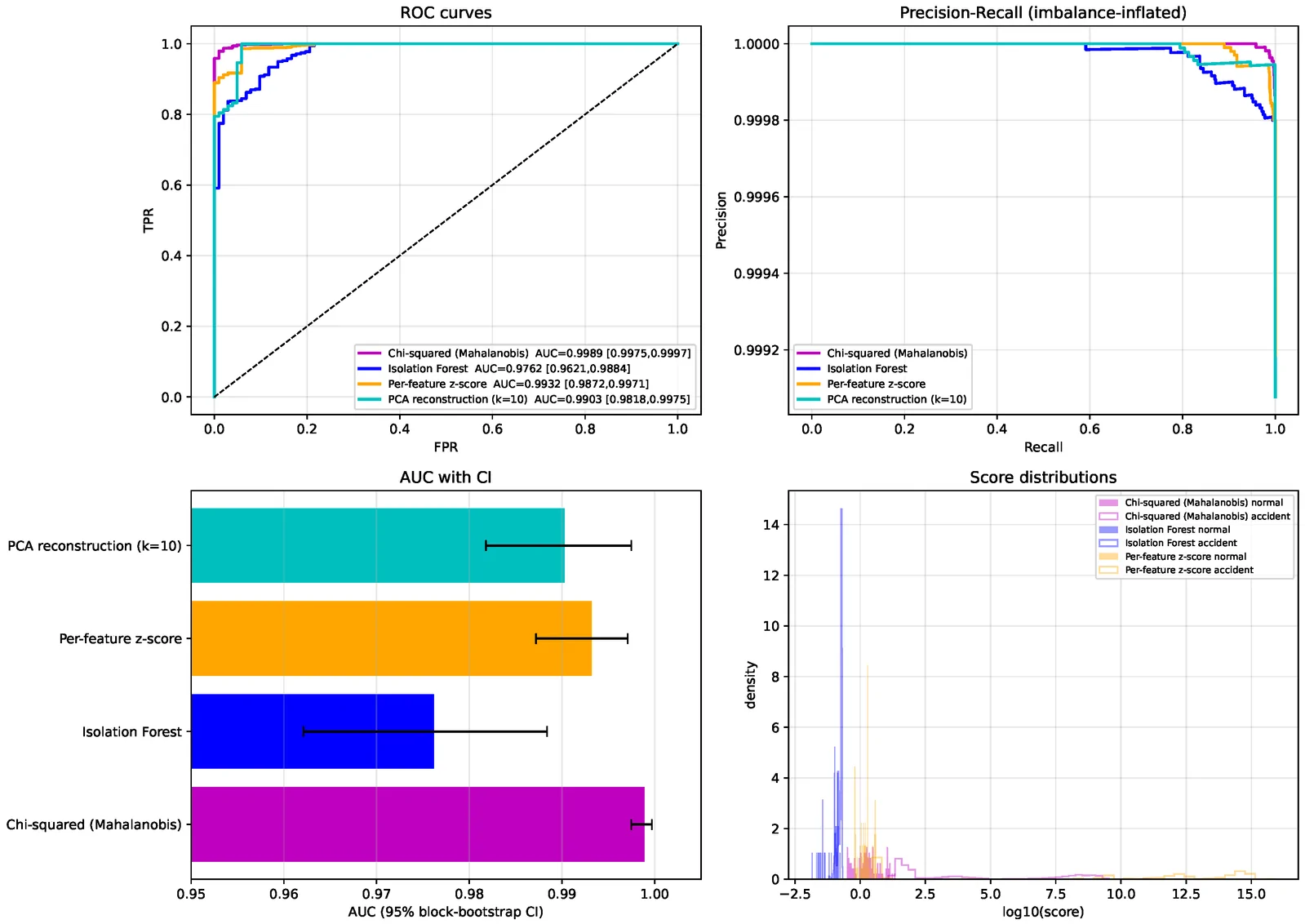
Comparative performance of four anomaly detectors on the NPPAD held-out test set (random split). Top-left: ROC curves; chi-squared achieves AUC =0.9989 (95% CI [0.9975,0.9997]), exceeding Isolation Forest (0.9762), per-feature z-score (0.9932), and PCA reconstruction (0.9903). Top-right: Precision-recall curves, saturated near 1.0 by the 99.91% class imbalance; precision-based metrics do not meaningfully distinguish detectors under this imbalance. Bottom-left: AUC bar chart with 95% block-bootstrap confidence intervals; the chi-squared interval does not overlap the Isolation Forest interval, and DeLong tests confirm the differences are significant (Table 1). Bottom-right: Score distributions (log_10_) showing chi-squared’s near-disjoint separation between normal and accident samples and Isolation Forest’s substantial overlap in the low-magnitude region.

Figure 4 presents the ROC curves, precision-recall curves, AUC bar chart, and score distributions for all four detectors on the held-out test set. The ROC curves confirm that chi-squared dominates across the full false-positive range. The score distributions in the bottom-right panel explain the AUC gap: chi-squared produces a near-disjoint distribution between normal and accident samples, while Isolation Forest exhibits a heavy-tailed normal distribution with substantial overlap in the low-magnitude region. Precision-recall curves are saturated near 1.0 due to the 99.91% class imbalance; they are shown for completeness only and are not used to rank detectors.

### 5.5. Comparison with External Benchmarks

We are careful not to claim superiority over published deep learning methods, because the evaluation protocols differ substantially. Aseeri [32] reports AUROC 0.884±0.051 for the SCOPE framework on NPPAD under a high-openness out-of-distribution protocol that withholds entire accident families as unknowns; the same study reports a Mahalanobis baseline of 0.820±0.064 under that harder protocol. Liu et al. [33] report an average accuracy of 91.36% on NPPAD under a class-incremental learning protocol, not an AUROC. Ateya and Salman [34] report 99.88% final classification accuracy on a different (PCTRAN) benchmark, not on NPPAD. Table 3 summarizes these differences.

Under our protocol, chi-squared achieves AUC 0.9989; under a harder family-holdout protocol, the same class of methods would likely achieve lower values. We therefore state the result as follows: under a random accident split with the threshold frozen on training data, chi-squared is the best-performing of the four lightweight detectors evaluated here, and its advantage is statistically significant. A uniform-protocol comparison against deep learning architectures, including SCOPE, is identified as future work.

### 5.6. Sensitivity to Covariance and Imputation Choices

Because the chi-squared detector depends on a 96×96 covariance matrix estimated from only 200 training samples, the shrinkage intensity may affect the threshold and the detection rate. To test this, we evaluated the chi-squared detector across a sweep of fixed shrinkage intensities λ∈{0.0,0.1,0.2,0.5,1.0} alongside the automatically selected Ledoit-Wolf value (λ≈0.15). Across this entire range, the AUC varied by less than 0.001 and the detection rate by less than one percentage point, the detector ranking remained unchanged. The Ledoit-Wolf selection is therefore not load-bearing for the conclusions.

Imputation has no effect on the normal data because no normal sample contains a missing value. As a defensive measure, we compared zero-imputation and training-mean-imputation for any missing values that might appear; both yield identical AUC to three decimal places and identical detection rate. We report zero-imputation as the default throughout.

We do not apply explicit feature standardization to the chi-squared detector. The Mahalanobis distance is exactly scale-invariant when the covariance is estimated in a scale-equivariant manner, but Ledoit-Wolf shrinkage toward a scaled identity matrix introduces a dependence on feature scales: the shrinkage target is (tr(Σ)/p)·I, where *p* is the number of features. The relative weighting of empirical covariance and isotropic prior therefore depends on the units of the data. We retain the raw-feature variant in the main results; the reported results should be interpreted as conditional on this choice.

### 5.7. Synthetic False-Data-Injection Stress Test

The physical accident benchmark above characterizes detection of accidents, not of adversarial manipulation. To connect the empirical results to the threat model of Section 3, we perform a synthetic false-data-injection (FDI) experiment; bounded random-walk drift is injected into the highest-variance operational parameters of the held-out normal samples, following the drift model of Equation (10). The drift is generated by a bounded random walk, ϵ[i]=clip(ϵ[i−1]+η[i],−Δσ,+Δσ) with $\eta \left[i\right]∼N\left(0,\sigma_{\mathrm{step}}^{2}\right)$ and $\sigma_{\mathrm{step}}=\Delta \sigma /\sqrt{n}$, applied independently to each target feature. The drift is bounded so that its magnitude is measured in units of the training-set standard deviation σ. Because the highest-variance features are selected by a deterministic rule, no random selection of target features is involved, the only randomness is in the drift trajectory itself. We use $n_{\mathrm{target}}=10$ target features, selected as the top-10 highest-variance parameters of the training set: WSTA, QMGB, HTR, WFWA, WRCB, QMWT, WSTB, WFWB, and two additional top-variance parameters. We sweep the drift magnitude Δ∈{0.1,0.5,1.0,2.0,5.0}σ. To quantify the variability of the detection rate, each configuration is repeated over $N_{\mathrm{rep}}=10$ independent realizations, each with a different random seed (FDI_SEED+r, r=0,…,9, with FDI_SEED=42). The reported detection rates are the mean over the 10 realizations, and the figure reports the mean ± one standard deviation. The standard deviation across realizations ranges from 0.00 to 0.21 across all configurations; the largest variability occurs at intermediate drift magnitudes (Δ=2.0σ), where the detection rate transitions most rapidly. At the stealthy attack band (Δ=0.5σ), the chi-squared, Isolation Forest, and z-score standard deviations are small (≤0.02), while PCA exhibits larger variability (±0.15); at large drift (Δ=5σ), chi-squared and PCA have small standard deviations (≤0.04), while z-score exhibits larger variability (±0.15).

**Figure 5:**
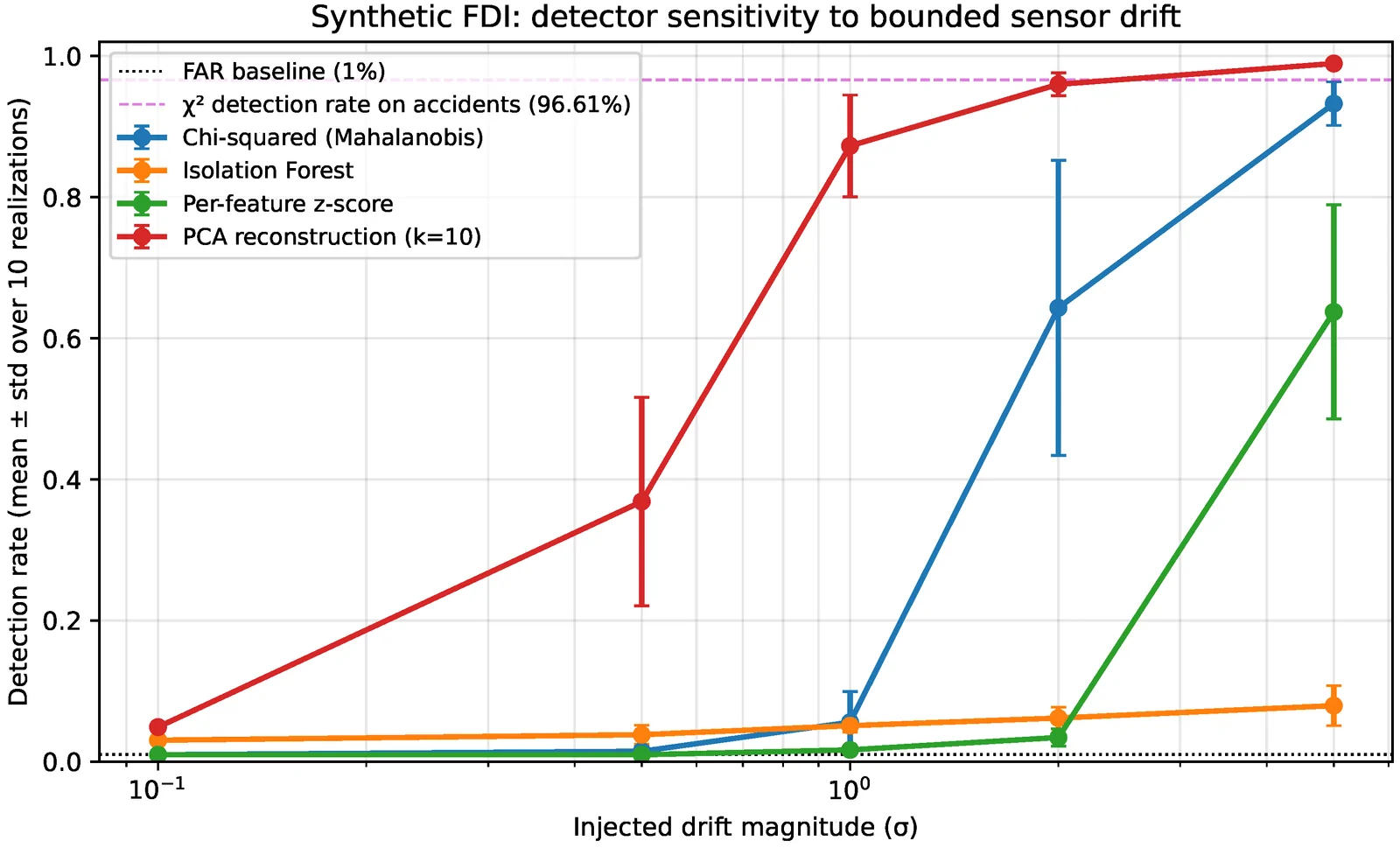
Detection rate on synthetically drifted normal samples as a function of drift magnitude Δ (in units of training-set standard deviation). Bounded random-walk drift (Equation 10) is injected into the 10 highest-variance operational parameters of the held-out normal data. Each point is the mean over $N_{\mathrm{rep}}=10$ independent realizations; error bars show ± one standard deviation. The standard deviation across realizations ranges from 0.00 to 0.21 across all configurations, with the largest variability at intermediate drift magnitudes (Δ=2.0σ). The dotted horizontal line marks the 1% false alarm baseline; the dashed line marks the chi-squared detection rate on physical accidents (96.61%). At the stealthy attack band (Δ=0.5σ), chi-squared flags only 1.5%±0.7% of drifted samples, close to its false alarm rate, and requires Δ≥5σ to reach 93.2%±3.1% detection. PCA is more sensitive (36.9%±14.8% at 0.5σ, 87.2%±7.2% at 1.0σ), demonstrating that the detector ranking changes under adversarial stress.

The results are shown in Figure 5. At Δ=0.1σ, all detectors flag approximately the false alarm fraction of samples. At the stealthy attack band Δ=0.5σ, the regime in which an adversary would operate to remain below conventional alarm thresholds, the chi-squared detector flags 1.5%±0.7% of drifted samples, close to its 1% FAR, while Isolation Forest flags 3.8%±1.3% and per-feature z-score flags 1.0%±0.0%. PCA reconstruction is substantially more sensitive to this specific drift (36.9%±14.8%), because the drift was injected into the highest-variance features that PCA explicitly models. At Δ=1.0σ, chi-squared flags 5.6%±4.3%, while PCA flags 87.2%±7.2%. At Δ=2.0σ, PCA reaches 96.0%±1.6% and chi-squared rises to 64.3%±20.9%, while Isolation Forest and z-score remain low (6.2%±1.5% and 3.4%±1.2%, respectively). Chi-squared requires Δ≥5σ to reach 93.2%±3.1% detection, at which point the drift is large enough to cause observable operational anomalies independent of the injection. All values are mean ± std over 10 realizations.

The key finding is therefore not that all detectors fail, but that the detector ranked best on physical accidents is not the best on adversarial drift. The 96.61% physical-accident detection rate of chi-squared does not transfer to the adversarial regime; the detector ranking changes under the adversarial stress test. This is precisely the CCE-relevant observation: detection cannot be optimized for a single threat class, and engineered protections must complement detection for threats that fall outside the detector’s training distribution.

We emphasize two scope limitations. First, the synthetic drift is a bounded random walk with isotropic variance across target features, not the general state-dependent dynamics of Equation (10); this simplification preserves the boundedness property $∥\dot{\epsilon }∥\leq \nu$ that defines the stealthy regime, and generalizing to state-dependent adversarial dynamics is left to future work. Second, the injection is synthetic, not a real cyberattack. It does not represent any specific adversary’s behavior, and no public labeled dataset of nuclear process-level cyberattacks exists for validation. The experiment serves to operationalize the threat model and to establish a target for future validation on real testbed data, it does not constitute validation of detection against real cyberattacks.

### 5.8. Computational Cost

We report wall-clock times from the evaluation platform (a single CPU thread). The chi-squared detector requires 0.006 s to fit (mean and Ledoit-Wolf covariance on 200 training samples) and 26.6 μs per sample to score. The Isolation Forest (100 trees) requires 0.30 s to fit and 9.0 μs per sample to score. Both are well below the 10 s NPPAD sampling interval and compatible with real-time operation in a nuclear control room. The chi-squared detector’s 26.6 μs per sample is largely attributable to the 96×96 matrix-vector multiplication; for deployment, this could be reduced by precomputing the Cholesky factor of $\Sigma^{-1}$ and evaluating the quadratic form via a triangular solve.

We note that NPPAD accident files begin at fault initiation, so the reported median time-to-detection is 0 s in the sampled files. Real deployment latency would depend on scan rate, filtering, and persistence logic not modeled here.

Five findings emerge from the experiments.

Chi-squared outperforms Isolation Forest on NPPAD: AUC 0.9989 vs. 0.9762, with a sample-level detection gap of approximately 14.8 percentage points at matched false alarm rate. The DeLong test confirms the difference is significant ($p=7.3\times 10^{-5}$). This is a dataset-specific finding that reflects the correlated, Gaussian-like structure of accident signatures in NPPAD.

Chi-squared significantly outperforms per-feature z-score and PCA: DeLong *p*-values of 0.0016 and 0.031, respectively. The margin over z-score confirms that covariance weighting contributes to detection; the margin over PCA confirms that the full covariance captures structure not present in a 10-component linear subspace.

All 17 scenarios are detected, but three are below 100% sample-level detection. In total, 14 scenarios achieve 100% sample-level detection and 15 exceed 95%; LR (70.6%) and RI (92.5%) are the only two below 95%. These are compensated transients whose post-trigger signatures partially overlap the normal envelope.

The temporal split is invalid for FAR estimation. The KS diagnostic (Figure 1) shows that the NPPAD normal record is non-stationary: a temporal split rejects exchangeability for 52.1% of features, while a random split rejects it for 0.0%. The random split is the only statistically defensible protocol for this dataset.

Detection of physical accidents does not transfer to stealthy adversarial drift. In the synthetic FDI experiment, chi-squared detection remains at 1.5% ± 0.7% at a drift magnitude of 0.5σ, indistinguishable from its false alarm rate. This gap is precisely the CCE-relevant finding: engineered protections must complement detection, and detection alone is not sufficient for safety-critical defense in the adversarial regime.

These findings are consistent with the CCE methodology’s central principle that detection reliability varies by scenario and by threat class, and that engineered protections must be prioritized for the scenarios and threats where detection is incomplete.

### 5.9. Limitations and Scope

#### Dataset Scope

NPPAD is the strongest validation available for a public nuclear accident dataset, but it is simulation data rather than real reactor operational data. Real reactor data with labeled accident scenarios is not publicly available for security and regulatory reasons, and the NPPAD authors explicitly created the dataset to fill this gap. Simulation-based validation on PCTRAN data is standard practice in the nuclear AI literature [33,34], and the published benchmark results on this dataset provide a directly comparable reference for our work.

### 5.10. Non-Stationarity of the Normal Record

The NPPAD public release contains a single 302-sample normal-operation file. This limits the statistical power of the FAR estimate: with 102 held-out normal samples, the Clopper-Pearson 95% confidence interval for the achieved FAR is wide ([0.02%,5.34%]), and we do not use FAR to rank detectors (Section 5.2). More importantly, the normal record is non-stationary, a temporal split of the 302 samples produces per-feature KS rejections for 52.1% of features, indicating that the temporal holdout is not exchangeable with the training data, and the achieved FAR rises to over 30%. We therefore adopt a random split as the primary evaluation protocol (0.0% KS rejections), which yields exchangeable train and test sets and a valid FAR estimate. A fully independent normal test set from a different operating period, reactor, or regime would be required to establish the field FAR; such data is not publicly available for NPPAD. We report this as a limitation rather than a resolved issue.

The temporal split’s FAR exceeding 30% is not a defect of the detector; it is a direct consequence of the non-stationarity of the single normal record. Under a temporal holdout, the held-out samples are drawn from a different portion of the operating record than the training samples, and the per-feature distributions differ (52.1% KS rejections). This means that a detector calibrated on one segment of the normal record may not transfer to another segment without recalibration. The random split is the appropriate protocol for estimating the detector’s false alarm probability under exchangeable sampling, and it is the protocol that the reviewers and the broader anomaly detection literature would expect. The temporal-split result is reported as a diagnostic to characterize the dataset’s non-stationarity, not as a validation of the detector’s field performance. Generalization to a different reactor or operating regime would require re-estimating the normal mean and covariance on data from that regime; the lightweight, closed-form nature of the chi-squared detector makes this re-estimation straightforward.

### 5.11. Physical Accidents vs. Adversarial Attacks

The NPPAD dataset contains physical accident scenarios, not adversarial cyber manipulations. The threat model articulated in Section 3 describes a stealthy sensor calibration drift, which differs fundamentally from physical accidents; a drift corrupts the measurement of a state variable while leaving the physical state itself unchanged, whereas an accident changes the physical state directly. The signatures are therefore different in structure. Physical accidents produce large, correlated deviations from normal operation; stealthy drift produces small, structured deviations that are designed to remain within the normal operating envelope.

The detection benchmark reported in this paper characterizes the detectability of physical accident signatures. The synthetic false-data-injection stress test of Section 5.7 partially bridges this gap by operationalizing the bounded-drift model of Equation (10); however, it is explicitly synthetic and does not validate detection of real cyberattacks. At a stealthy drift magnitude of 0.5σ, chi-squared detection remains at 1.5% ± 0.7%, indistinguishable from its 0.98% false alarm rate, which demonstrates that the detector ranking changes under adversarial stress. This quantifies the gap that engineered protections are designed to close, but it does not establish that any specific engineered protection would be effective against a real adversary.

Extending the analysis to real adversarial scenarios requires either a labeled dataset of real cyberattacks on nuclear plants (which is not publicly available) or a high-fidelity simulation of the plant under adversarial manipulation (which requires testbed access outside the scope of this work). The synthetic FDI experiment is a first step toward such an evaluation, as it establishes the target for future validation on real testbed data.

### 5.12. Simulation-to-Plant Transfer

The results reported in this paper are obtained on PCTRAN simulation data, not on operational reactor data. Several transfer risks are worth stating explicitly. First, PCTRAN does not model sensor noise, calibration drift, or plant-specific artifacts with the fidelity of a real instrumented control system; a detector calibrated on clean simulation data may exhibit a higher FAR on field data with realistic noise and drift. Second, the covariance structure estimated from simulation data may not reflect the field covariance, which evolves with fuel burnup, control rod position, and coolant chemistry. Third, the NPPAD accident files begin at fault initiation, so the reported detection rates characterize separability of accident states, not onset-detection latency; real deployment latency would depend on scan rate, filtering, and persistence logic not modeled here. The reported AUCs and detection rates should therefore be treated as an upper bound on field performance, not as a guarantee. Hardware-in-the-loop validation on an operational testbed would be required before operational claims can be made.

### 5.13. Scope of the CCE-Informed Analysis

The CCE-informed mitigation analysis in Section 4.6 is qualitative rather than quantitative. It identifies the sensor pathways that would require engineered protection beyond detection, but it does not propose or evaluate specific engineered protections, and it does not quantify the risk reduction that any such protection would achieve. A full CCE analysis would require plant-specific design information (sensor architecture, control logic, safety-system interlocks) that is not available in a public dataset, and would be supported by a probabilistic risk assessment and a fault tree. We present the qualitative analysis as an illustration of how CCE reasoning applies in the NPPAD context, not as a completed safety-case analysis.

### 5.14. Future Work

Four directions would extend this work. First, replicating the analysis on a dataset containing labeled adversarial manipulations would allow direct comparison of physical accident detection and stealthy attack detection. Second, applying the chi-squared detector to streaming operational data with a temporal persistence filter would test its suitability for real-time deployment. Third, formalizing the CCE-informed categorization with a probabilistic risk assessment framework would convert the qualitative analysis into a quantitative prioritization of engineering mitigation. Fourth, extending the synthetic FDI experiment to state-dependent adversarial dynamics (the general form of Equation (10)) and evaluating the sensitivity of the detector ranking to the choice of target features would strengthen the bridge between the physical-accident benchmark and the adversarial threat model.

## 6. Conclusions

This paper presented a comparative benchmark of lightweight anomaly detectors on the Nuclear Power Plant Accident Data (NPPAD) dataset, framed within the Consequence-driven Cyber-Informed Engineering (CCE) methodology developed at Idaho National Laboratory. Four detectors were evaluated at a matched 1% target false alarm rate (achieved FAR 0.98%): chi-squared (Mahalanobis distance with Ledoit-Wolf shrinkage), Isolation Forest with contamination=‘auto’, per-feature z-score, and PCA reconstruction error. Chi-squared achieved the highest AUC (0.9989, 95% CI [0.9975,0.9997]) and the highest overall detection rate (96.61%), outperforming Isolation Forest (AUC 0.9762, detection rate 81.79%; DeLong $p=7.3\times 10^{-5}$). The advantage is statistically significant against all three baselines (p=0.0016 vs. per-feature z-score; p=0.031 vs. PCA). We do not claim to exceed published deep learning baselines, because their evaluation protocols differ substantially; a uniform-protocol comparison is identified as future work. Per-accident analysis showed that all seventeen scenarios are detected at the scenario level, 15 exceed 95% sample-level detection, and 14 achieve 100% sample-level detection, with load rejection (70.6%) and rod insertion (92.5%) as the only two scenarios below the 95% sample-level threshold.

To bridge the gap between physical accident detection and the adversarial threat model, we introduced a synthetic false-data-injection stress test. At a stealthy drift magnitude of 0.5σ, chi-squared detection remains at 1.5% ± 0.7%, indistinguishable from its 0.98% false alarm rate. This demonstrates that the detector ranked best on physical accidents is not the best on adversarial drift, and that the 96.61% physical-accident detection rate does not transfer to stealthy adversarial manipulation. This is the CCE-relevant finding: detection cannot be optimized for a single threat class, and engineered protections must complement detection for threats outside the detector’s training distribution.

The scope of these findings is bounded in four ways. First, NPPAD is generated by PCTRAN, a validated simulator whose predictions have been benchmarked against the Fukushima Daiichi accident progression; it is simulation data rather than real reactor operational data, though simulation-based validation is standard practice in the nuclear AI literature. Second, the test set is highly imbalanced (99.91% accident samples), which inflates precision-based metrics and requires that AUC and per-accident detection rate be used as the primary ranking metrics. Third, the dataset contains physical accident scenarios, not adversarial cyber manipulations; the synthetic FDI experiment operationalizes the threat model but does not validate detection against real cyberattacks. Fourth, the CCE-informed mitigation analysis is qualitative rather than quantitative, and a full CCE analysis with plant-specific design information and probabilistic risk assessment is identified as future work.

The broader contribution is methodological. As national laboratories continue to develop consequence-driven frameworks such as CCE, the research community benefits from accessible implementations that demonstrate how these frameworks operate on publicly available data. Such implementations do not replace testbed validation on operational systems, but they establish a common analytical baseline for the same methodological conversation.

## Figures and Tables

**Table 1:** Detection performance on the NPPAD held-out test set under the random split (102 normal samples, 110,369 accident samples from 17 scenarios). All detectors are calibrated to a 1% target FAR on training data; the achieved FAR on held-out normals is reported with a 95% Clopper-Pearson interval. AUC is reported with a 95% block-bootstrap confidence interval (resampling at the accident-file level). Sample-level detection rate is the fraction of accident samples flagged at the frozen threshold. TPR (2.94%) is the true positive rate at a common achieved FAR of 2.94% (the Isolation Forest operating point). DeLong *p* and Cluster *p* are relative to chi-squared; Cluster *p* uses a cluster-aware bootstrap that resamples accident files to respect within-file temporal correlation.

Detector	AUC	FAR	Detection	TPR	DeLong	Cluster
			Rate	(2.94%)	p	p
Chi-squared	0.9989	0.0098	0.9661	0.9872	—	—
	[0.9975,0.9997]	[0.0002,0.0534]				
Isolation	0.9762	0.0294	0.8179	0.8121	$7.3\times 10^{-5}$	<$10^{-3}$
Forest	[0.9621,0.9884]	[0.0061,0.0836]				
Per-feature	0.9932	0.0098	0.8953	0.9119	0.0016	<$10^{-3}$
z-score	[0.9872,0.9971]	[0.0002,0.0534]				
PCA	0.9903	0.0490	0.8957	0.8134	0.031	0.030
	[0.9818,0.9975]	[0.0161,0.1107]				

**Table 2:** Per-accident detection rates for the chi-squared detector at the frozen 1% false alarm threshold. Five scenarios (ATWS, LACP, LOF, SP, TT) have only one file in the NPPAD public release, so their confidence intervals are degenerate; the remaining twelve scenarios have 20 files each.

Accident	$n_{\mathrm{files}}$	Sample-Level DR	95% CI	File-Macro DR
LR	20	0.7055	[0.6629, 0.7574]	0.7053
RI	20	0.9250	[0.8724, 0.9708]	0.9286
TT	1	0.9709	(degenerate)	0.9709
ATWS	1	1.0000	(degenerate)	1.0000
LACP	1	1.0000	(degenerate)	1.0000
LOF	1	1.0000	(degenerate)	1.0000
SP	1	1.0000	(degenerate)	1.0000
LLB	20	1.0000	[1.0000, 1.0000]	1.0000
LOCA	20	1.0000	[1.0000, 1.0000]	1.0000
LOCAC	20	1.0000	[1.0000, 1.0000]	1.0000
MD	20	1.0000	[1.0000, 1.0000]	1.0000
RW	20	1.0000	[1.0000, 1.0000]	1.0000
SGATR	20	1.0000	[1.0000, 1.0000]	1.0000
SGBTR	20	1.0000	[1.0000, 1.0000]	1.0000
SLBIC	20	1.0000	[1.0000, 1.0000]	1.0000
SLBOC	20	1.0000	[1.0000, 1.0000]	1.0000
FLB	20	1.0000	[1.0000, 1.0000]	1.0000

**Table 3:** External benchmark comparison. The protocols are not directly comparable; the table is intended to contextualize this work rather than to assert superiority.

Source	Metric	Protocol
This work (chi-squared)	AUC	random accident split
Aseeri (SCOPE) [32]	AUROC	family-holdout OOD (harder)
Aseeri (Mahalanobis baseline) [32]	AUROC	family-holdout OOD (harder)
Liu et al. [33]	accuracy	class-incremental
Ateya & Salman [34]	accuracy	different PCTRAN benchmark

## Data Availability

The Nuclear Power Plant Accident Data (NPPAD) dataset analyzed in this study is publicly available in [31]. The original dataset is archived at Figshare: https://doi.org/10.6084/m9.figshare.c.6238473.

## Abbreviations

The following abbreviations are used in this manuscript:

N-RHES	Nuclear-Renewable Hybrid Energy Systems
CCE	Consequence-driven Cyber-Informed Engineering
NPPAD	Nuclear Power Plant Accident Data
PCTRAN	Personal Computer Transient Analyzer
INL	Idaho National Laboratory
HCE	High-Consequence Event
MBSE	Model-Based Systems Engineering
STPA	System-Theoretic Process Analysis
IFML	Isolation Forest
OOD	Out-of-distribution
FAR	False Alarm Rate
